# Bone Induction as a Function of Size and Chemical Composition of Calcium Phosphate Granules in Osteogrow-C Evaluated in Animal Models: A 1-Year Follow-Up

**DOI:** 10.34133/bmr.0337

**Published:** 2026-02-25

**Authors:** Nikola Stokovic, Natalia Ivanjko, Marina Milesevic, Katarina Muzina, Marko Pecin, Drazen Maticic, Slobodan Vukicevic

**Affiliations:** ^1^ Laboratory for Mineralized Tissues, School of Medicine, University of Zagreb, Zagreb, Croatia.; ^2^ Genera Research, Kalinovica, Croatia.; ^3^ Department of Inorganic Chemical Technology and Non-metals, Faculty of Chemical Engineering and Technology, University of Zagreb, Zagreb, Croatia.; ^4^ Clinics for Surgery, Orthopedics and Ophthalmology, Faculty of Veterinary Medicine, University of Zagreb, Zagreb, Croatia.

## Abstract

Calcium phosphate (CaP) ceramics are widely used in bone regenerative medicine for their osteoconductive properties. Osteogrow-C is a novel device that comprises recombinant human bone morphogenetic protein 6 (rhBMP6) in autologous blood coagulum, utilizing ceramics as a compression-resistant matrix. This study evaluated how CaP granule size and composition affect bone formation and implant integrity in 2 relevant animal models: the rat subcutaneous model and rabbit posterolateral lumbar fusion (PLF) model, over 1 year. The implants in the rat model had varying granule size ranges (74 to 420 μm, 500 to 1,700 μm, 2,360 to 4,000 μm) and compositions [β-tricalcium phosphate (β-TCP), hydroxyapatite (HA), and biphasic ceramics (TCP/HA 80/20)]. Micro-computed tomography (CT) and histology showed that Osteogrow-C induced bone formation on all ceramic scaffolds, with smaller granules resulting in higher bone volume and density, regardless of composition. TCP granules were most resorbed, but residual ceramics persisted in all groups. Based on these findings, Osteogrow-C, containing small granules with different compositions (TCP, HA, TCP/HA 80/20, and TCP/HA 40/60), was further tested in the clinically relevant rabbit PLF model and induced fusion of transverse processes. Importantly, ceramics were more resorbed in the rabbit PLF model, with TCP and TCP/HA 80/20 ceramics showing the highest resorption rate, while HA remained intact. Osteogrow-C containing HA showed increased bone volume; however, biomechanical strength and thicker cortical bone were achieved with TCP and biphasic calcium phosphate (BCP). Finally, in the rat model, bone volume was primarily dependent on granule size, with smaller granules promoting greater bone formation and density. Conversely, in the PLF model, the composition played a more important role—affecting ceramic resorption, bone volume, and biomechanical properties.

## Introduction

Bone tissue has remarkable regenerative abilities; however, its natural capacity is often inadequate to heal larger defects. Autologous bone graft (ABG), harvested from the iliac crest, has excellent osteoinductive, osteoconductive, and osteogenic properties and is considered the gold standard for treating large bone defects [1]. Moreover, ABG is also used to achieve spinal fusion in patients suffering from various degenerative spinal diseases [2]. Nevertheless, due to its numerous drawbacks and limited availability, there is an imminent need for the development of autologous bone graft substitutes (ABGSs).

Calcium phosphate (CaP) synthetic ceramics are widely used for bone regeneration due to their excellent osteoconductive properties [3]. The most commonly used CaP materials include β-tricalcium phosphate [β-TCP; Ca₃(PO₄)₂] and hydroxyapatite [HA; Ca₁₀(PO₄)₆(OH)₂], which greatly differ in their resorbability: TCP is more resorbable, while HA is very stable [4]. Moreover, TCP and HA can be combined into biphasic calcium phosphate (BCP) to adjust the resorbability of ceramics [5]. Osteoconductive CaP ceramics can be combined with potent osteoinductive molecules such as bone morphogenetic proteins (BMPs). Although CaP ceramics have been extensively employed as BMP carriers or compression-resistant matrices (CRM) in numerous preclinical studies, it remains unclear whether TCP or HA is superior in this setting. Therefore, ceramics made from TCP [6–8], HA [9,10], and BCP with different TCP/HA ratios [11–14] formulated in different sizes and shapes were used in previous studies. However, only a few in vivo studies have focused on the comparison between TCP and HA to promote bone induction [15–17], and their results were inconsistent. Moreover, previous studies have predominantly focused on evaluating various osteoinductive devices during osteogenesis over short periods of time following bone formation [6–18]. Additionally, although it is known that smaller ceramic granules are superior to larger granules in bone induction, it is not clear how the size of ceramic granules affects the long-term outcome and microarchitecture of induced ectopic bone.

Osteogrow is a novel ABGS containing recombinant human bone morphogenetic protein 6 (rhBMP6) delivered within autologous blood coagulum (ABC) as a BMP carrier [19]. CRM may be added to ABC to improve the biomechanical properties of implants [20–22]. Efficacy and safety of Osteogrow and formulations with the addition of allograft (Osteogrow-A) or synthetic CaP ceramics (Osteogrow-C) were previously demonstrated in a series of preclinical studies [rabbit and sheep posterolateral fusion (PLF) model, rabbit segmental bone defect model] [20,21,23–27] and clinical trials (high-tibial osteotomy, distal radius fracture, PLF) [28,29]. Although both allograft and CaP ceramics promote bone formation [20–22,26,30–33], CaP ceramics are the preferred option because they overcome major allograft disadvantages, including the risk of viral transmission and immunogenicity [34].

The key question when defining the formulation of Osteogrow-C implants for clinical use is the selection of the optimal size and composition of ceramic granules. It has been previously demonstrated that, following a short period (21 d after implantation), TCP and HA granules combined with rhBMP6/ABC are equally efficient in promoting bone induction. Additionally, Osteogrow-C implants with small granules induce larger amounts of bone than those containing larger granules [22]. However, it remains unclear how the chemical composition and size of granules affect bone microarchitecture over an extended period, and which material is preferable for use as a CRM in an osteoinductive device. To address this gap, the present study employed a unique long-term study design with assessment at a single 1-year (365-d) endpoint and 2 complementary animal models—a rat subcutaneous assay and a rabbit PLF model—providing a comprehensive understanding of material behavior across different biological environments. Specifically, the aim was to determine how the chemical composition of ceramic granules used as CRM with Osteogrow (rhBMP6/ABC) influences the structure of newly formed bone and ceramic resorption over time, and whether these effects differ between a subcutaneous ectopic site in rats and a functionally active site in rabbit posterolateral spinal fusion.

## Materials and Methods

### Experimental design

**Fig. 1.**
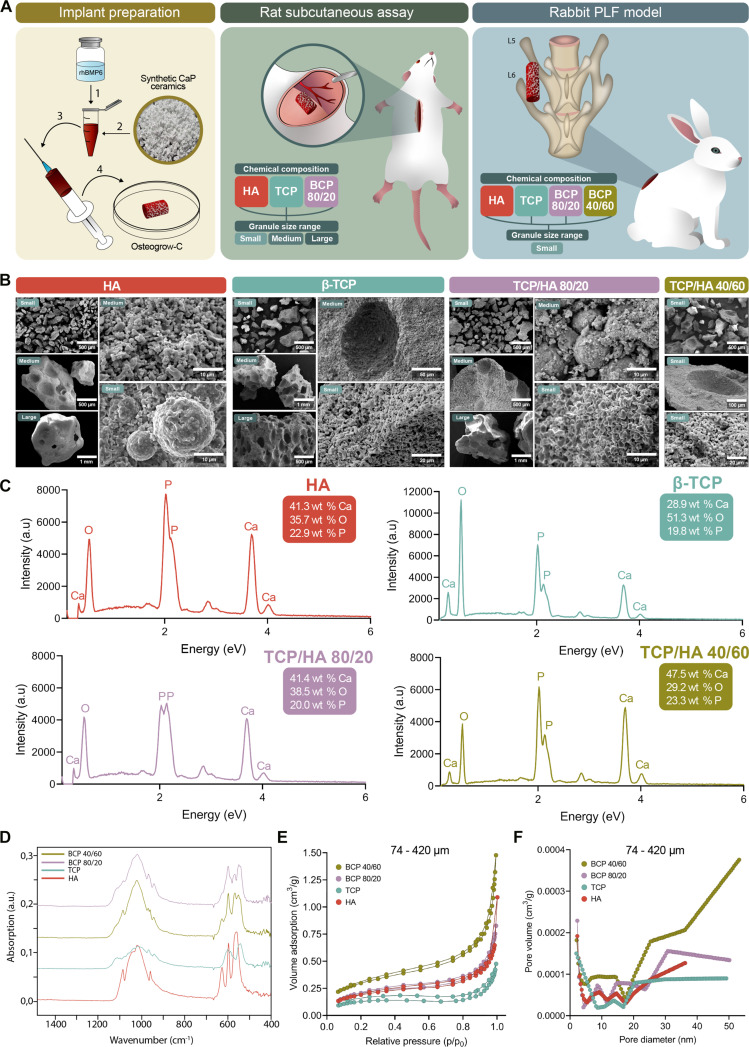
Experimental design and characterization of synthetic CaP ceramics as CRM in Osteogrow-C. (A) Experimental design of the study, presenting a schematic image of implant preparation that involves combining rhBMP6 with autologous blood and various types of ceramic granules. Osteogrow-C implants were evaluated in vivo using 2 animal models: a rat subcutaneous implantation model with ceramics of various chemical compositions (HA, biphasic TCP/HA 80/20, and β-TCP) and granule size ranges (74 to 420 µm, 500 to 1,700 µm, and 2,360 to 4,000 µm), and a rabbit posterolateral fusion (PLF) model in which selected ceramic compositions and granule sizes were tested for spinal fusion between transverse processes. (B) Morphological analysis by scanning electron microscopy (SEM) revealed the surface features and granule morphology of hydroxyapatite (HA), β-tricalcium phosphate (β-TCP), and biphasic ceramics (TCP/HA 80/20 and TCP/HA 40/60), with microstructural variations observed across different granule sizes. (C) Chemical composition was assessed using energy-dispersive x-ray spectroscopy (EDS), demonstrating the elemental composition of each ceramic formulation by quantifying calcium, phosphorus, and oxygen content. (D) Fourier transform infrared (FTIR) spectroscopy further characterized these materials by highlighting the distinct vibrational bands associated with each ceramic group. (E) Nitrogen adsorption–desorption isotherms (BET) were used to analyze differences in surface area and pore characteristics among the small ceramic granules of different chemical compositions, while (F) pore size distribution analysis confirmed distinct micro- and mesoporous structures across the various ceramic groups.

To compare the effect of the chemical composition of synthetic ceramic granules used as CRM in Osteogrow-C implants on the long-term microarchitecture and longevity of newly formed bone between a subcutaneous ectopic site and a functionally active site, we conducted 2 experiments using a rat subcutaneous assay and a rabbit PLF model (Fig. 1A).

In the rat subcutaneous experiment, we tested 3 different chemical compositions of granules (TCP, HA, and biphasic ceramics containing TCP and HA in an 80/20 ratio) in 3 different sizes (74 to 420 μm, 500 to 1,700 μm, and 2,360 to 4,000 μm) (Table 1). Based on the results of this experiment, we selected small granules (74 to 420 μm), which yielded the highest bone volume, for further testing in the rabbit posterolateral spinal fusion model. In the rabbit PLF experiment, we compared small granules in 4 different chemical compositions (TCP, TCP/HA 80/20, TCP/HA 40/60, and HA) (Table 1). The synthetic ceramic granules were produced by CaP BioMaterials (East Troy, WI, USA), while rhBMP6 was produced by Genera Research (Zagreb, Croatia) as previously described [27]. The outcomes in both experiments were evaluated 365 d after implantation. The number of specimens per group was 6 in both experiments.

**Table 1.** Experimental design of the study

Rat subcutaneous assay	
A	rhBMP6/ABC + HA 74–420 μm
B	rhBMP6/ABC + HA 500–1,700 μm
C	rhBMP6/ABC + HA 2,360–4,000 μm
D	rhBMP6/ABC + TCP/HA (80/20) 74–420 μm
E	rhBMP6/ABC + TCP/HA (80/20) 500–1,700 μm
F	rhBMP6/ABC + TCP/HA (80/20) 2,360–4,000 μm
G	rhBMP6/ABC + TCP 74–420 μm
H	rhBMP6/ABC + TCP 500–1,700 μm
I	rhBMP6/ABC + TCP 2,360–4,000 μm
Rabbit PLF model	
A	rhBMP6/ABC + TCP 74–420 μm
B	rhBMP6/ABC + TCP/HA (80/20) 74–420 μm
C	rhBMP6/ABC + TCP/HA (40/60) 74–420 μm
D	rhBMP6/ABC + HA 74–420 μm

### Evaluation of the structural and physical attributes of synthetic ceramic granules

Prior to the evaluation of Osteogrow-C implants in animal models, a comprehensive characterization and comparison of synthetic CaP ceramics (CaP Biomaterials, East Troy, WI, USA) was conducted. Namely, HA, β-TCP, and 2 BCPs with TCP/HA ratios of 80/20 and 40/60 in various size ranges were thoroughly characterized using scanning electron microscopy (SEM), energy-dispersive x-ray spectroscopy (EDS), Fourier transform infrared spectroscopy (FTIR), and physisorption analysis based on nitrogen adsorption–desorption isotherms. Furthermore, all tested ceramic particles (across compositions and nominal size groups) were characterized by measuring the granule size distribution (Fig. S1).

The morphology of the samples was evaluated by SEM. Sample preparation involved mounting the specimens onto aluminum stubs using double-sided carbon conductive tape, followed by sputter coating with a gold–palladium alloy using a Quorum SC7620 coater (Quorum Technologies, East Sussex, UK) to enhance conductivity. SEM imaging was performed on a Tescan Vega 3EasyProbe SEM (Tescan, Brno, Czech Republic) operated at 10-kV accelerating voltage. Elemental analysis was carried out with an integrated Bruker B-Quantax EDS system. Fourier transform infrared (FTIR) spectra were recorded using a Bruker Vertex 70 spectrometer (Bruker Optics, Ettlingen, Germany) in attenuated total reflectance (ATR) mode, with a spectral resolution of 2 cm^−1^ and averaging 32 scans per sample. Physisorption analysis was performed exclusively on small ceramic granules (74 to 420 μm) since the method requires samples to fit into small capillary tubes for accurate gas adsorption analysis at cryogenic temperatures. Nitrogen adsorption–desorption isotherms for all samples were recorded at 77 K using an ASAP 2000 instrument (Micromeritics, Norcross, GA, USA), with prior degassing at 100 °C under dynamic vacuum (7 mPa) to remove physically adsorbed species. The specific surface area was calculated using the Brunauer–Emmett–Teller (BET) method, and the pore size distribution was derived from the desorption branch of the isotherm using the Barrett–Joyner–Halenda (BJH) model.

### Experimental animals

The rat subcutaneous implant experiments were performed on Sprague–Dawley laboratory rats (lat. *Rattus norvegicus*, male, 6 to 10 weeks old, weighing 200 to 300 g), while the rabbit PLF experiment was carried out on New Zealand White rabbits (lat. *Oryctolagus cuniculus*, male, 15 weeks old, weighing 3,000 to 3,500 g). The experimental animals were housed in the animal facility within a standard, carefully monitored laboratory environment (temperature 20 to 24 °C, relative humidity 40% to 70%, 12 h of light per day, and noise levels of 60 dB). Standard good laboratory practice (GLP) diet and fresh water were provided ad libitum. Approval for the studies was given by the Directorate for Veterinary and Food Safety, Ministry of Agriculture, Republic of Croatia, following evaluation of the Ethics Committee at the University of Zagreb School of Medicine and the National Ethics Committee (EP 187/2018, EP 191/2019, and EP 296/2020).

### Osteogrow-C implant preparation and surgical procedures

Synthetic ceramic granules (0.1 g in the rat experiment and 0.8 g in the rabbit experiment) were placed in sterile syringes according to the experimental design of the study (Table 1). Blood (0.5 ml in the rat experiment and 2.5 ml in the rabbit experiment) was collected into tubes without anticoagulant, and rhBMP6 (20 μg in the rat experiment and 125 μg in the rabbit experiment) was added to the blood. The blood was then drawn into syringes, gently mixed, and left to coagulate. All implants were implanted within 1 h of preparation. Laboratory rats were anesthetized with a combination of xylazine (5 mg/kg, intramuscularly) and ketamine (100 mg/kg, subcutaneously) before the surgical procedure. Osteogrow-C implants were implanted into subcutaneous pockets in the axillary region, as described previously [22]. The posterolateral spinal fusion experiment was conducted in 12 New Zealand White rabbits as previously described [20,26]. In brief, the transverse processes of lumbar vertebrae L5 and L6 were exposed and decorticated using a high-speed burr. Osteogrow-C implants were implanted bilaterally, and the fascial/skin incisions were closed with 3-0 synthetic glycolide/lactide copolymer absorbable sutures. Experimental animals in both experiments were euthanized 365 d after implantation. Following euthanasia, rat implants and rabbit lumbar spine segments were extracted and fixed in 10% formalin for further analysis.

### Micro-CT analysis

Newly formed bone and fused spinal segments were scanned using the 1076 SkyScan μCT device (Bruker, Kontich, Belgium) to quantify the amount of bone and remaining CRM, as well as to assess bone microarchitecture. The scanning resolution was 18 μm, with frame averaging of 2 and a rotation step of 0.5°. The reconstruction of the acquired images was conducted using NRecon software (Bruker, Kontich, Belgium) with appropriate beam hardening correction to minimize artefacts. Further bone assessment and analysis were performed using CTAn software (Bruker, Kontich, Belgium), which was used to calculate the volume of bone and CRM, CRM surface, as well as trabecular parameters (trabecular thickness, trabecular separation, and trabecular number) of the new bone. Segmentation of newly formed bone and residual CRM particles was achieved through density-based thresholding [23], exploiting the characteristic grayscale intensity differences between materials. CaP ceramics typically exhibit higher x-ray attenuation coefficients than trabecular or woven bone due to their greater mineral density and crystalline structure, resulting in distinct grayscale ranges that facilitate automated segmentation. Global threshold values were determined for each material type and applied consistently across all specimens within the study to ensure reproducible quantification.

### Histology

The obtained samples were fixed in 10% formalin for 10 d and processed either decalcified (rat subcutaneous experiment) or undecalcified (rabbit PLF experiment and selected specimens from the rat experiment), as described previously [22,30]. In brief, most rat samples were decalcified using 14% EDTA in 4% formalin solution for 20 d, and after decalcification, the specimens were embedded in paraffin. Rabbit spine specimens and selected rat specimens were polymerized into hardened acrylic resin blocks following dehydration in graded solutions of ethyl alcohol. Finally, 5-μm sections were obtained, mounted on gelatin-coated glass slides, and stained with Goldner, Von Kossa, and hematoxylin and eosin.

### Histomorphometry

Histomorphometric analysis was conducted to quantify the proportions of bone, bone marrow, and residual ceramic granules in histological sections obtained 1 year after subcutaneous implantation. Sections stained with Goldner’s trichrome were used for this evaluation. Representative slides from each experimental group were imaged at a consistent magnification of 10× using an Olympus BX53 microscope coupled with cellSens Dimension software (Olympus, Tokyo, Japan). Bone and ceramic surfaces in the digital images were segmented and color-coded using Photoshop (Adobe Systems, San Jose, CA, USA). Subsequent quantification of these delineated regions was performed with Fiji ImageJ software (version 1.51r, NIH, Bethesda, MD, USA), with the bone marrow area calculated indirectly by subtracting the areas occupied by bone and ceramic granules from the total area. Results were presented as pie charts to illustrate the percentage distribution of bone, bone marrow, and residual ceramic granules within each experimental group. Alongside relative percentages, absolute bone area (mm^2^) was also reported. Additionally, interparticle distances (mm) between residual ceramic granules were measured in each image, with a minimum of 10 measurements per image to ensure representative sampling. Cortical thickness (mm) of newly formed bone between the 2 transverse processes in the rabbit PLF model was measured using CellSens Dimension software.

### Biomechanical testing

A 3-point bending test was conducted using a TA.HDplus instrument (Stable Micro Systems, Godalming, UK) to determine the biomechanical parameters (maximum force, elasticity, and work-to-break) of the newly formed bone in the rabbit PLF experiment. The fusion mass, comprising newly formed bone integrated with native transverse processes, was placed on 2 supports, and force was applied perpendicular to the midpoint as previously described [26].

### Data analysis

The normality of all datasets was assessed utilizing the Kolmogorov–Smirnov test. For data exhibiting a Gaussian distribution, comparisons among 3 or more groups were conducted using one-way analysis of variance (ANOVA) followed by Tukey’s post hoc test for multiple comparisons. Kruskal–Wallis test, accompanied by Dunn’s multiple comparisons post hoc test, was used for non-normally distributed data. To explore the relationships among the variables and to reduce data dimensionality, Spearman’s rank correlation and principal components analysis (PCA) were performed. Spearman’s rank correlation coefficient was calculated to assess the strength and direction of monotonic associations between variables. For pairwise (2-variable) comparisons, Spearman correlation coefficients were presented as a line to illustrate the strength and direction of the relationship. When exploring correlations among multiple variables, the results were visualized as a heatmap to effectively convey the pattern and magnitude of associations within the data matrix. PCA was performed to identify the main axes of variation within the dataset and to reduce the dimensionality while preserving the maximum amount of variance. The number of components retained is determined by the Kaiser rule, retaining those with eigenvalues greater than 1. Results were presented as either mean ± standard deviation or as median along with minimum and maximum values, as appropriate. Findings were regarded as statistically significant when *P* was less than 0.05. Statistical significance was indicated by asterisks, where *P ≤ 0.05, **P ≤ 0.01, and ***P ≤ 0.001. The statistical analyses were performed using RStudio (Posit PBC, Boston, MA, USA) and GraphPad Prism (Dotmatics, Boston, MA, , USA).

## Results

### Distinct morphological, elemental, and porosity profiles of CaP ceramics as CRM component in Osteogrow-C device

Before in vivo evaluation (Fig. 1A), different CaP ceramics intended as the CRM component of Osteogrow-C were thoroughly characterized for their morphology, elemental composition, and porosity profiles. Morphological analysis using a SEM revealed distinct structural features among the different ceramics (Fig. 1B). HA presented as a compact material with densely packed, irregular, crystalline-like structures. In HA samples, medium-sized granules (500 to 1,700 μm) tended to exhibit a more granular or irregular surface texture, whereas small granules predominantly displayed smoother, spherical morphologies. In contrast, in TCP/HA 80/20, the medium-sized granules were typically more spherical, whereas the small granules were more irregular and grainier. On the other hand, β-TCP ceramics were characterized by their rough, irregular, and highly porous surface architecture, featuring numerous interconnected pores and channels. Across all experimental groups, sieve analysis confirmed that over 95% of the particles were within the nominal size range (Fig. S1).

Chemical composition, as determined by EDS, also varies across the ceramics (Fig. 1C). HA and TCP/HA 40/60 displayed the highest calcium content (41.3 and 47.5 wt %, respectively). In contrast, β-TCP was distinguished by reduced levels of calcium (28.9 wt %) and substantially higher oxygen fraction (51.3 wt %) compared to HA. TCP/HA 80/20 was closely aligned with HA, showing only minimal deviations in the main elemental proportions. Notably, TCP/HA 40/60 exhibited the most pronounced divergence, characterized by the highest calcium content of all samples (47.5 wt %), moderate phosphorus (23.3 wt %), and the lowest oxygen percentage (29.2 wt %). EDS provides only semiquantitative data from the near-surface region, so results must be interpreted with caution. Based on the obtained weight fractions of calcium and phosphorus, the Ca/P molar ratios of each sample were calculated. HA showed a ratio of 1.39 (versus theoretical 1.67), indicating surface calcium deficiency, while β-TCP had 1.13 (versus 1.50), suggesting phosphorus enrichment. Biphasic ceramics gave intermediate values: TCP/HA 80/20 = 1.60 and TCP/HA 40/60 = 1.58, both higher than theoretical β-TCP. These results reflect heterogeneous Ca and P distribution, consistent with the coexistence of HA and TCP phases. These variations in elemental composition underscore the purposeful tuning of these biomaterials, from the relatively inert and slowly resorbing HA, through the more reactive and resorbable β-TCP, to the custom properties of the biphasic formulations.

Fourier transform infrared (FTIR) spectroscopy analysis confirmed the characteristic bands corresponding to individual PO₄^3−^ vibrational modes in similar spectral regions across all samples (Fig. 1D). Notably, HA samples displayed both the characteristic PO₄^3−^ vibrational bands located at 562, 600, 962, 1,021, 1,044, and 1,088 cm^−1^, as well as a distinct OH^−^ group band at 629 cm^−1^, reflecting the presence of structural hydroxyl groups unique to HA. In contrast, TCP samples lacked the pronounced OH^−^ band and exhibited subtle shifts and broader features in the PO₄^3−^ modes, consistent with differences in crystal symmetry and chemical composition between these phases. The biphasic BCP 40/60 and BCP 80/20 samples exhibited additional bands for each PO₄^3−^ vibrational mode, along with the OH^−^ group band, indicative of the coexistence of both HA and TCP phases within their compositions. These spectral differences highlight the distinct structural characteristics of HA and TCP, with direct implications for their stability, solubility, and ion release profiles in biological environments.

Porosity and surface area, assessed by nitrogen adsorption, further distinguished the influence of their chemical composition among small granules (Fig. 1E). All samples exhibited type IV isotherms with H3 hysteresis loops (IUPAC classification), characteristic of mesoporous materials composed of particulate aggregates. The porosity measured falls within the mesoporous range (2 to 50 nm). TCP/HA 40/60 exhibited greater nitrogen adsorption, indicating the largest accessible surface area and the highest degree of porosity. Pore size distribution analysis reinforced these findings (Fig. 1F). Namely, TCP/HA 40/60 exhibited the highest pore volume, particularly at larger pore diameters (above 30 nm), indicating the presence of a substantial population of larger mesopores. Results of the BET specific surface areas and average pore diameter are presented in Table 2.

**Table 2.** BET specific surface area and average pore diameter of different calcium phosphate ceramics (HA, TCP, BCP 40/60, and BCP 80/20) evaluated as CRM components for Osteogrow-C

Sample	BET specific surface area (m^2^/g)	Average pore diameter (nm)
HA	0.7261	5.4225
TCP	0.4923	4.8281
TCP/HA 40/60	1.1136	6.7237
TCP/HA 80/20	0.8373	5.2841

### Ceramic size as the main determinant of structural properties of BMP-induced bone in rat subcutaneous assay

**Fig. 2.**
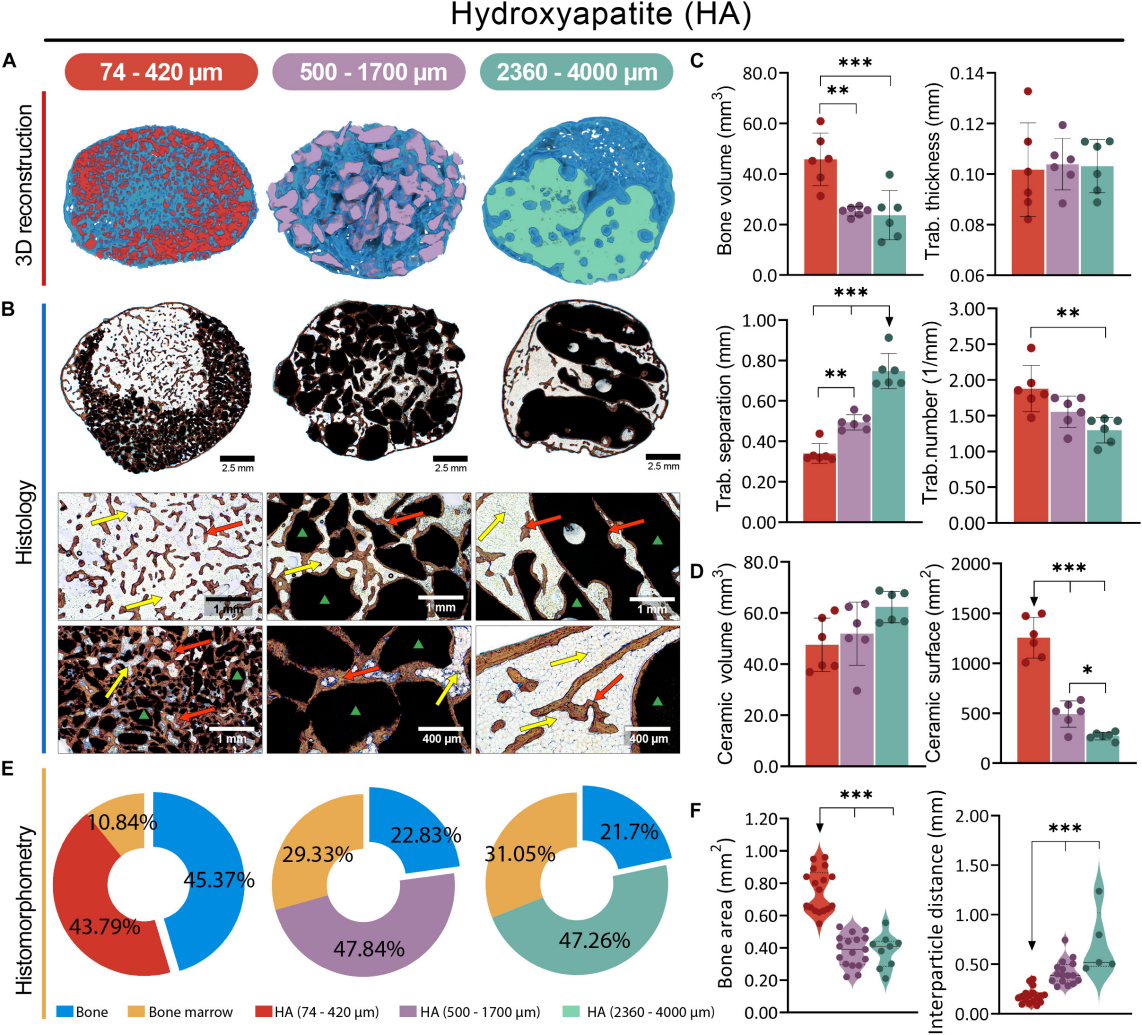
Evaluation of Osteogrow-C implants containing HA ceramic granules of different sizes—small: 74 to 420 μm (red), medium: 500 to 1,700 μm (purple), and large: 2,360 to 4,000 μm (green)—in a rat subcutaneous model 1 year post-implantation. (A) Micro-CT 3D and (B) histological cross sections of newly formed ectopic bone induced by Osteogrow-C containing HA in various sizes, stained by Sanderson’s Rapid Bone Stain with Van Gieson picrofuchsin. Red arrows indicate newly formed bone, yellow arrows denote adipocytic bone marrow, while the green triangle marks ceramic granules. Scale bars are shown in the lower right corner. (C) Micro-CT quantification of newly formed bone ossicles induced by Osteogrow-C implant showing bone volume (mm^3^), and trabecular parameters, thickness (mm), number (1/mm), and separation (mm). (D) Micro-CT quantification of volume (mm^3^) and surface area (mm^2^) of residual ceramic granules. The number of specimens analyzed per group was 6. (E) Pie chart representation of histomorphometric analysis showing the relative proportions (%) of bone (blue), adipocytic bone marrow (yellow), and residual ceramic granules. (F) Histomorphometric quantification of bone area (mm^2^) together with interparticle distance (mm). All *P* values below 0.05 were considered significant and are marked with asterisks: *P ≤ 0.05, **P ≤ 0.01, and ***P ≤ 0.001.

**Fig. 3.**
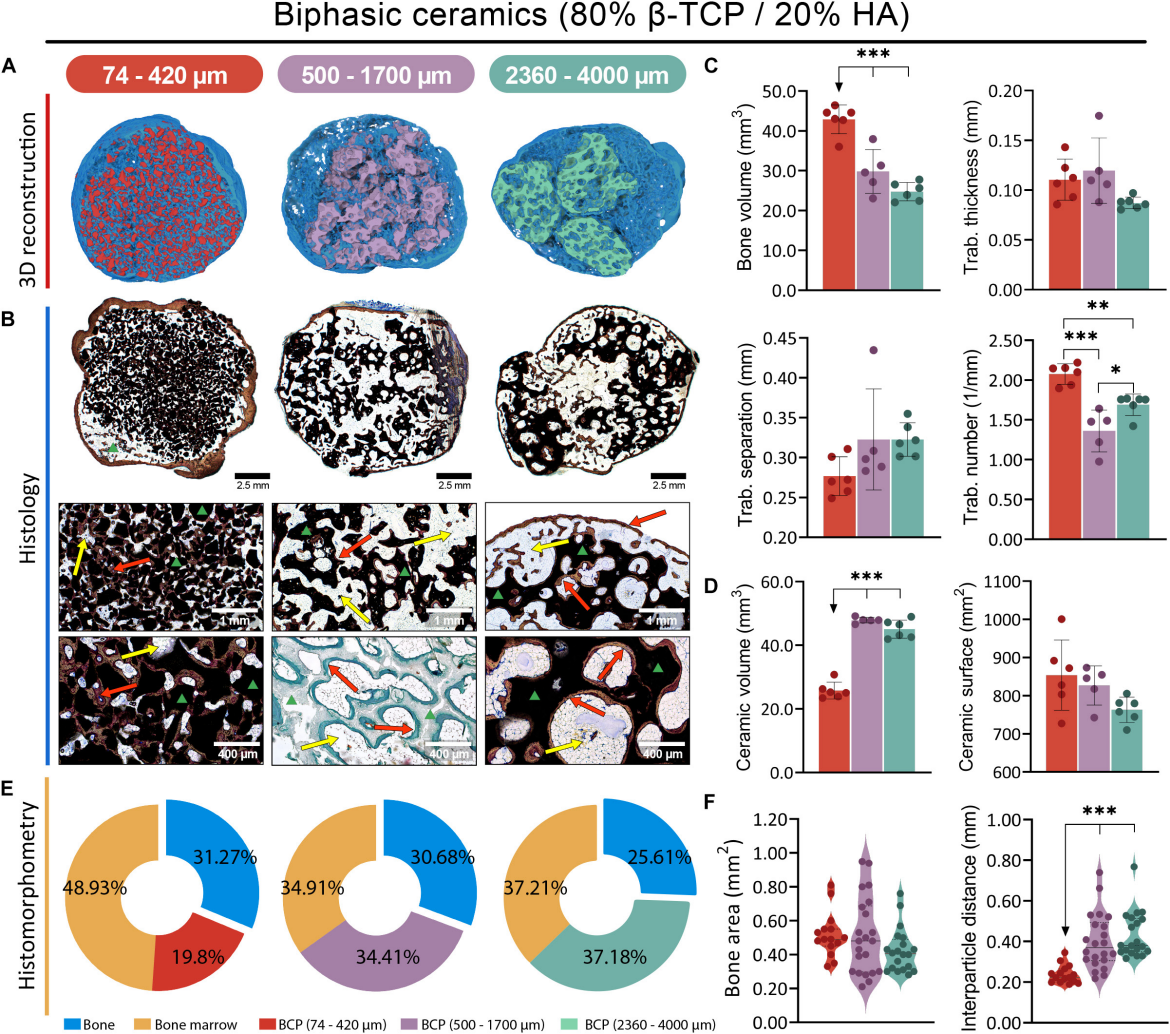
Evaluation of Osteogrow-C implants containing biphasic ceramic granules (80% β-TCP, 20% HA) of different sizes—small: 74 to 420 μm (red), medium: 500 to 1,700 μm (purple), and large: 2,360 to 4,000 μm (green)—in a rat subcutaneous model, 1 year after implantation. (A) Micro-CT 3D and (B) histological cross sections of newly formed ectopic bone induced by Osteogrow-C containing biphasic granules in various sizes, stained by Sanderson’s Rapid Bone Stain with Van Gieson picrofuchsin or modified Goldner Trichrome. Red arrows indicate newly formed bone, yellow arrows denote adipocytic bone marrow, while the green triangle marks ceramic granules. Scale bars are shown in the lower right corner. (C) Micro-CT quantification of newly formed bone ossicles induced by Osteogrow-C implant showing bone volume (mm^3^), and trabecular parameters, thickness (mm), number (1/mm), and separation (mm). (D) Micro-CT quantification of volume (mm^3^) and surface area (mm^2^) of residual ceramic granules. The number of specimens analyzed per group was 6. (E) Pie chart representation of histomorphometric analysis showing the relative proportions (%) of bone (blue), adipocytic bone marrow (yellow), and residual ceramic granules. (F) Histomorphometric quantification of bone area (mm^2^) together with interparticle distance (mm). All *P* values below 0.05 were considered significant and are marked with asterisks: *P ≤ 0.05, **P ≤ 0.01, and ***P ≤ 0.001.

**Fig. 4.**
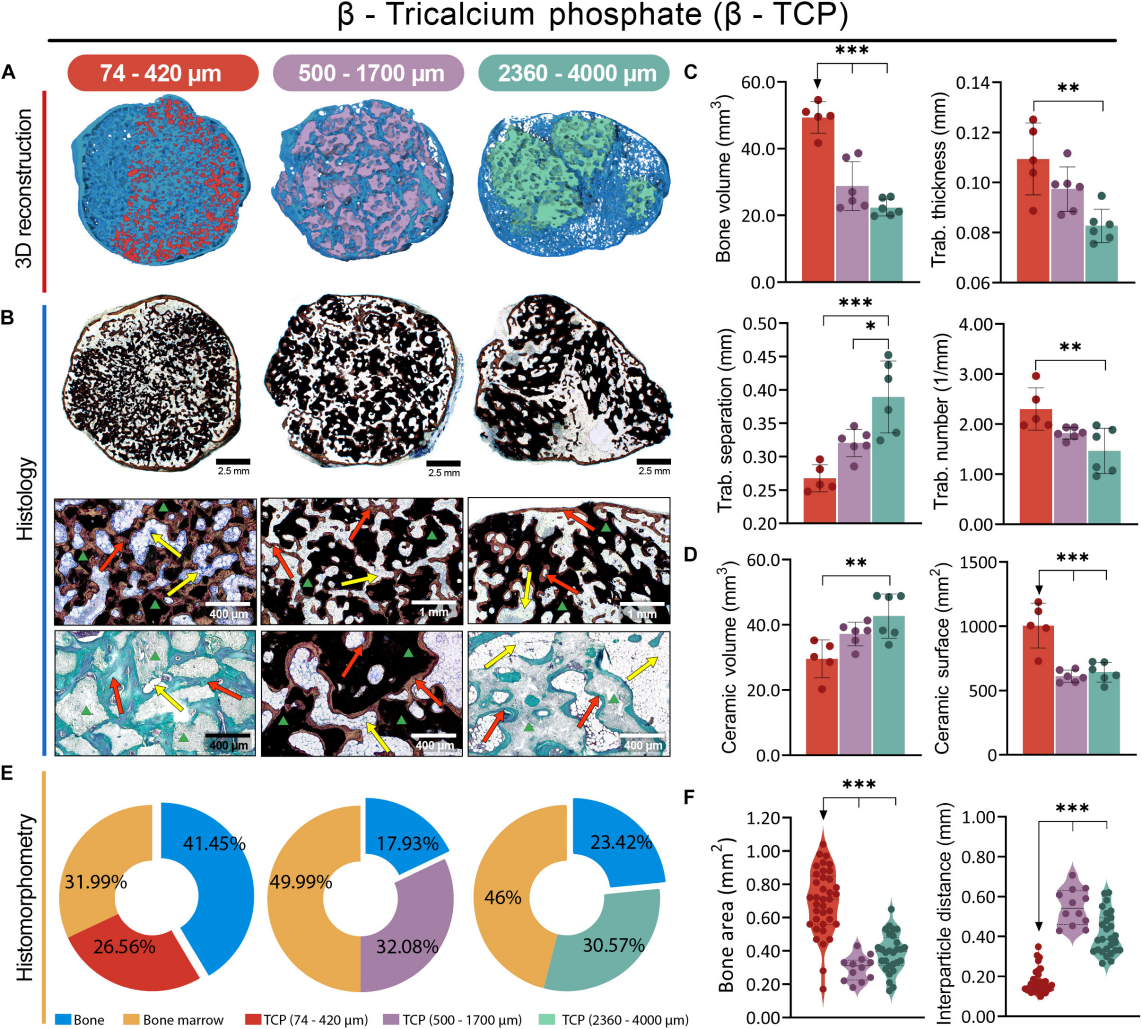
Evaluation of Osteogrow-C implants containing β-TCP ceramic granules of varying sizes—small: 74 to 420 μm (red), medium: 500 to 1,700 μm (purple), and large: 2,360 to 4,000 μm (green)—in a rat subcutaneous model, 1 year after implantation. (A) Micro-CT 3D and (B) histological cross sections of newly formed ectopic bone induced by Osteogrow-C containing β-TCP granules in various sizes, stained by Sanderson’s Rapid Bone Stain with Van Gieson picrofuchsin or modified Goldner Trichrome. Red arrows indicate newly formed bone, yellow arrows denote adipocytic bone marrow, while the green triangle marks ceramic granules. Scale bars are shown in the lower right corner. (C) Micro-CT quantification of newly formed bone ossicles induced by Osteogrow-C implant showing bone volume (mm^3^), and trabecular parameters, thickness (mm), number (1/mm), and separation (mm). (D) Micro-CT quantification of volume (mm^3^) and surface area (mm^2^) of residual ceramic granules. The number of specimens analyzed per group was 6. (E) Pie chart representation of histomorphometric analysis showing the relative proportions (%) of bone (blue), adipocytic bone marrow (yellow), and residual ceramic granules. (F) Histomorphometric quantification of bone area (mm^2^) together with interparticle distance (mm). All *P* values below 0.05 were considered significant and are marked with asterisks: *P ≤ 0.05, **P ≤ 0.01, and ***P ≤ 0.001.

**Fig. 5.**
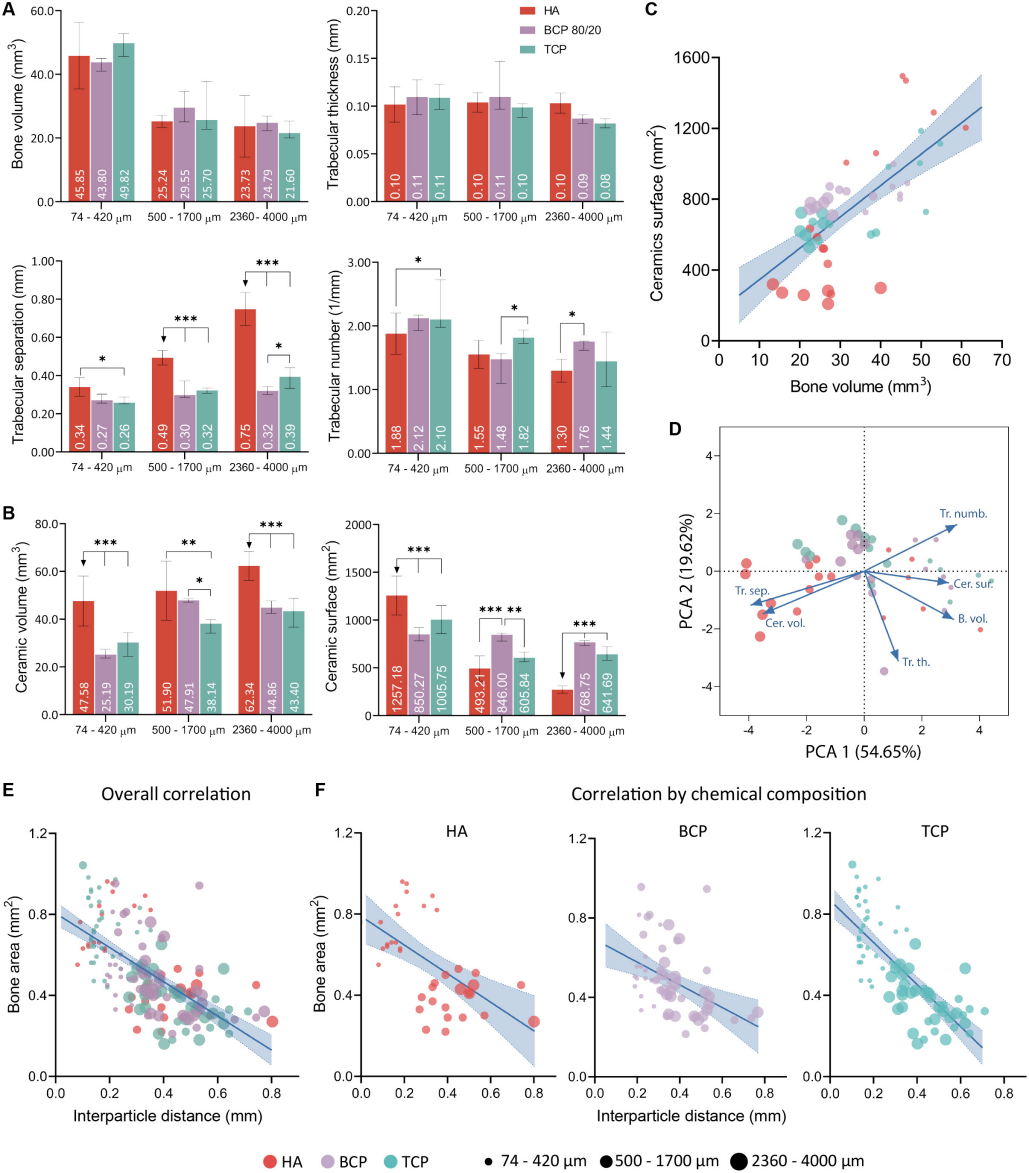
Characterization of bone formation as a function of biomaterial chemical composition and granule size. (A) Micro-CT quantitative analysis of new bone volume and remaining CRM volume of HA (red color), biphasic calcium phosphate (TCP/HA 80/20) (purple color), and TCP (green color) in 3 different granule size ranges. (B) Micro-CT quantification of volume (mm^3^) and surface area (mm^2^) of residual ceramic granules. (C) Spearman’s rank pairwise correlation of micro-CT evaluated the CRM surface (mm^2^) and bone volume (mm^3^). (D) Principal components analysis (PCA) illustrates clustering of experimental groups and their relationships to key bone parameters (bone volume, CRM volume and surface, trabecular thickness, number, and separation). (E) Overall negative correlation between distances (mm) among ceramic granules and bone area (mm^2^). (F) Correlation between particle distance (mm) and bone area (mm^2^) depicted separately for HA, BCP, and TCP groups, revealing similar negative trends for all compositions and particle sizes (indicated by point size and color).

All Osteogrow-C implants with HA (Fig. 2), BCP (Fig. 3), and TCP (Fig. 4) ceramics induced the formation of ectopic composite ossicles composed of newly formed bone and residual ceramic granules. The newly formed bone persisted throughout the 1-year period, while the residual ceramic volume was determined by the chemical composition of the ceramics (Figs. 2A and B, 3A and B, and 4A and B). However, the size of the ceramic granules was the primary determinant of the bone’s structural properties, resulting in striking differences among ossicles containing granules of different sizes (Figs. 2C to F, 3C to F, 4C to F, and 5A).

In ectopic composite ossicles containing small ceramic granules (74 to 420 μm), the bone was divided into 3 regions: bone present on the surfaces of the ceramic granules, bone between the ceramic granules, and cortical bone forming the boundaries of the ectopic ossicle (Figs. 2A and B, 3A and B, and 4A and B). Due to the uneven distribution of small ceramic granules within the implants, parts of the ectopic ossicle contained no ceramic granules (Fig. 2B, first column). In areas with ceramic granules, there were regions resembling compact bone and others resembling trabecular bone (Fig. 4B, first column). In the compact bone-like areas, the bone tissue was extremely dense, with continuous bone across the ceramic surfaces and very limited bone marrow. Conversely, in the trabecular bone-like areas, bone was present on the surfaces and between the ceramic granules, but large areas of bone marrow were also present. The distance between small ceramic granules was generally minimal (Figs. 2F, 3F, and 4F), with no significant differences in interparticle spacing observed between implant regions exhibiting these bone patterns. In areas without ceramic granules, trabecular bone was abundant, with widespread bone marrow and a few bone trabeculae (Fig. 2B, first column). Although the relative amount of bone marrow varied in different parts of the ossicle, its composition was consistent, with adipocytes being the predominant cells, followed by hematopoietic cells (Figs. 2B, 3B, and 4B).

Ossicles containing medium (500 to 1,700 μm) and large (2,360 to 4,000 μm) ceramic granules exhibited pronounced cortical bone at the boundaries, newly formed bone on both the inner and outer surfaces of the ceramic granules, and trabecular bone between them (Figs. 2A and B, 3A and B, and 4A and B, second and third columns). The primary difference between medium and large granules, aside from size, was the presence of a complex pore network inside the large granules (Figs. 2B, 3B, and 4B, third columns). In comparison, the medium granules had few pores (Figs. 2B, 3B, and 4B, second columns). Bone was consistently present in the pores, regardless of their location within the granules.

Bone formed on the inner ceramic surfaces encircled the pores, which contained bone marrow. The distance between adjacent medium and large ceramic granules was significantly larger than between small granules (Figs. 2F, 3F, and 4F), with trabeculae of bone connecting adjacent ceramic granules and surrounded by bone marrow containing adipocytes and only a few hematopoietic cells (Figs. 2B, 3B, and 4B). However, medium HA granules had flat surfaces, allowing closer contact between granules in some areas, where a dense bone network with limited bone marrow formed, similar to the compact bone-like areas in ossicles containing small granules (Fig. 2B, second column).

### Ceramic size determines bone volume, while chemical composition determines residual ceramic volume in the rat subcutaneous assay

Micro-computed tomography (micro-CT) and histomorphometric analyses revealed that all Osteogrow-C formulations induced the formation of ectopic composite ossicles containing extensive amounts of bone, which persisted for 365 d following implantation. However, bone volume (micro-CT parameter) and bone area (histomorphometric parameter) were highest in ossicles containing small granules (74 to 420 μm), followed by those with medium (500 to 1,700 μm) and large granules (2,360 to 4,000 μm), regardless of the chemical composition of the granules (Figs. 2C, E, and F; 3C, E, and F; 4C, E, and F; and 5A). These findings indicate that bone volume was determined by granule size rather than chemical composition. Furthermore, trabecular number was significantly higher, while trabecular separation was significantly lower in ossicles containing small granules compared to those with medium or large granules (Figs. 2C, 3C, 4C, and 5A). These trends were consistently observed across all experimental groups, regardless of the ceramic granules’ chemical composition. Furthermore, histomorphometric analyses further revealed that the interparticle distance was significantly greater in ossicles containing medium and large granules than in those with small granules (Figs. 2F, 3F, and 4F).

Residual CRM volume, as determined by micro-CT, was highest in ossicles containing pure HA granules and lowest in those with pure TCP granules, regardless of the granule size (Figs. 2D, 3D, 4D, and 5B). Ossicles with biphasic ceramics (TCP/HA 80/20) exhibited residual ceramic volumes similar to those observed in the TCP group. These results indicate that the residual ceramic volume was primarily influenced by the chemical composition of the ceramic granules, rather than size. However, it is important to note that in all groups, including those with HA granules, a considerable amount of nonresorbed ceramic material remained. A significant amount of nonresorbed granules correlates with the observation that, even after 1 year, small ceramic granules retained the largest surface area, irrespective of their chemical composition (Figs. 2D, 3D, 4D, and 5B).

PCA and correlation analyses were conducted to investigate the relationships between the variations in different micro-CT parameters across samples. Among the key findings was a positive correlation between the surface area of ceramic granules and the bone volume of newly formed tissue (Fig. 5C and D). In contrast, the correlation analysis of histomorphometric parameters revealed a negative correlation between bone area and the interparticle distance of ceramic granules (Fig. 5E and F).

### Ceramic resorption was significantly higher in the rabbit PLF model compared to the rat subcutaneous site, while the chemical composition of the ceramics was the main determinant of bone structural properties

**Fig. 6.**
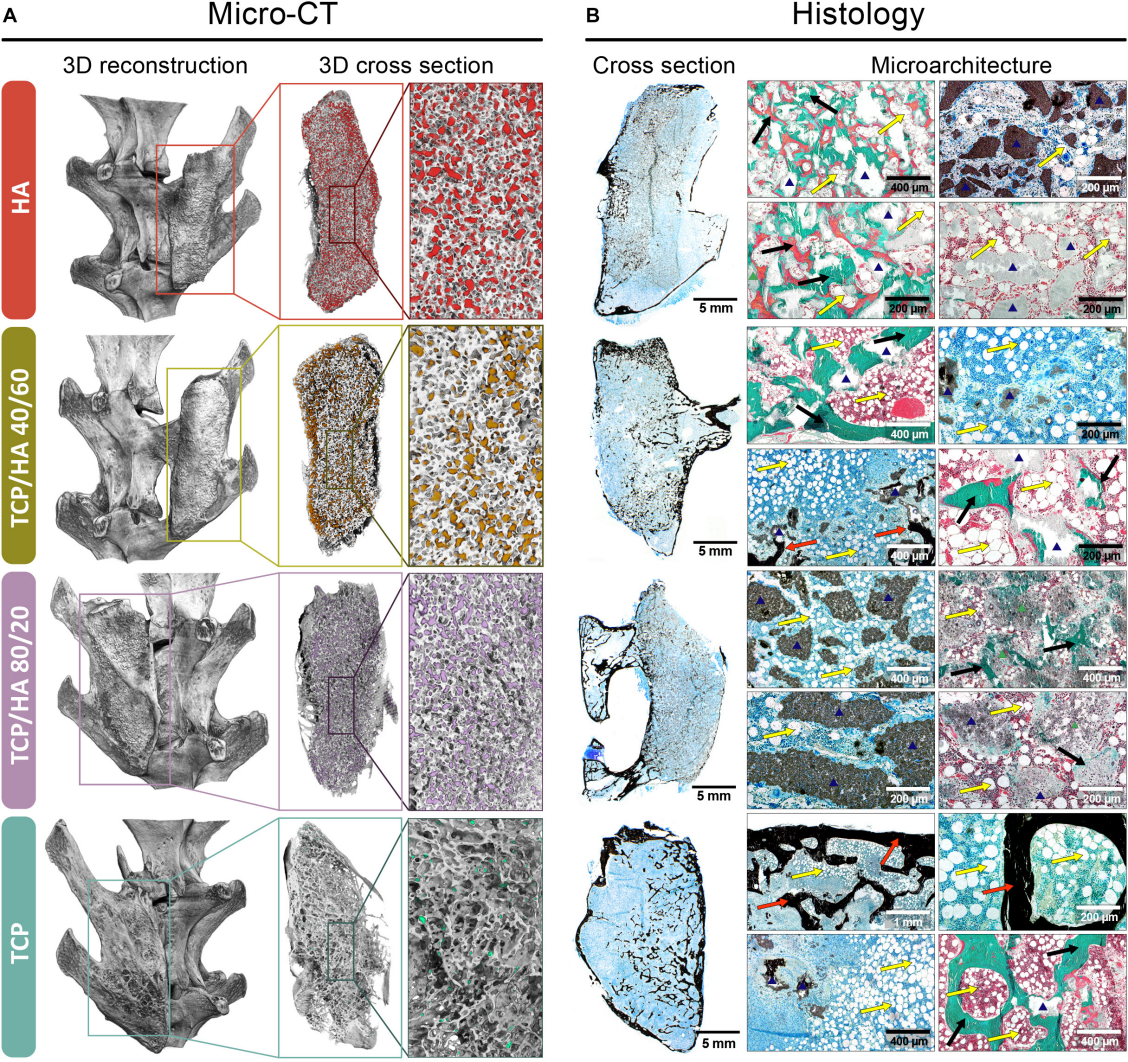
Micro-CT and histological analysis of bone induced by Osteogrow-C formulations containing small ceramic granules in various chemical compositions in a rabbit PLF model, 1 year following implantation. (A) Micro-CT 3D reconstructions and cross-sections depict the newly formed bone between the transverse processes following the application of different Osteogrow-C ceramic formulations, varying in chemical composition: HA, biphasic ceramics (TCP/HA 40/60 and TCP/HA 80/20), and β-TCP. (B) Corresponding histological cross-sections and microarchitecture images illustrate the quality and distribution of new bone, residual ceramic material, and connective tissue for each group. Black or red arrows indicate newly formed bone, yellow arrows denote adipocytic bone marrow, while the blue triangle marks ceramic granules. Scale bars are shown in the lower right corner.

**Fig. 7.**
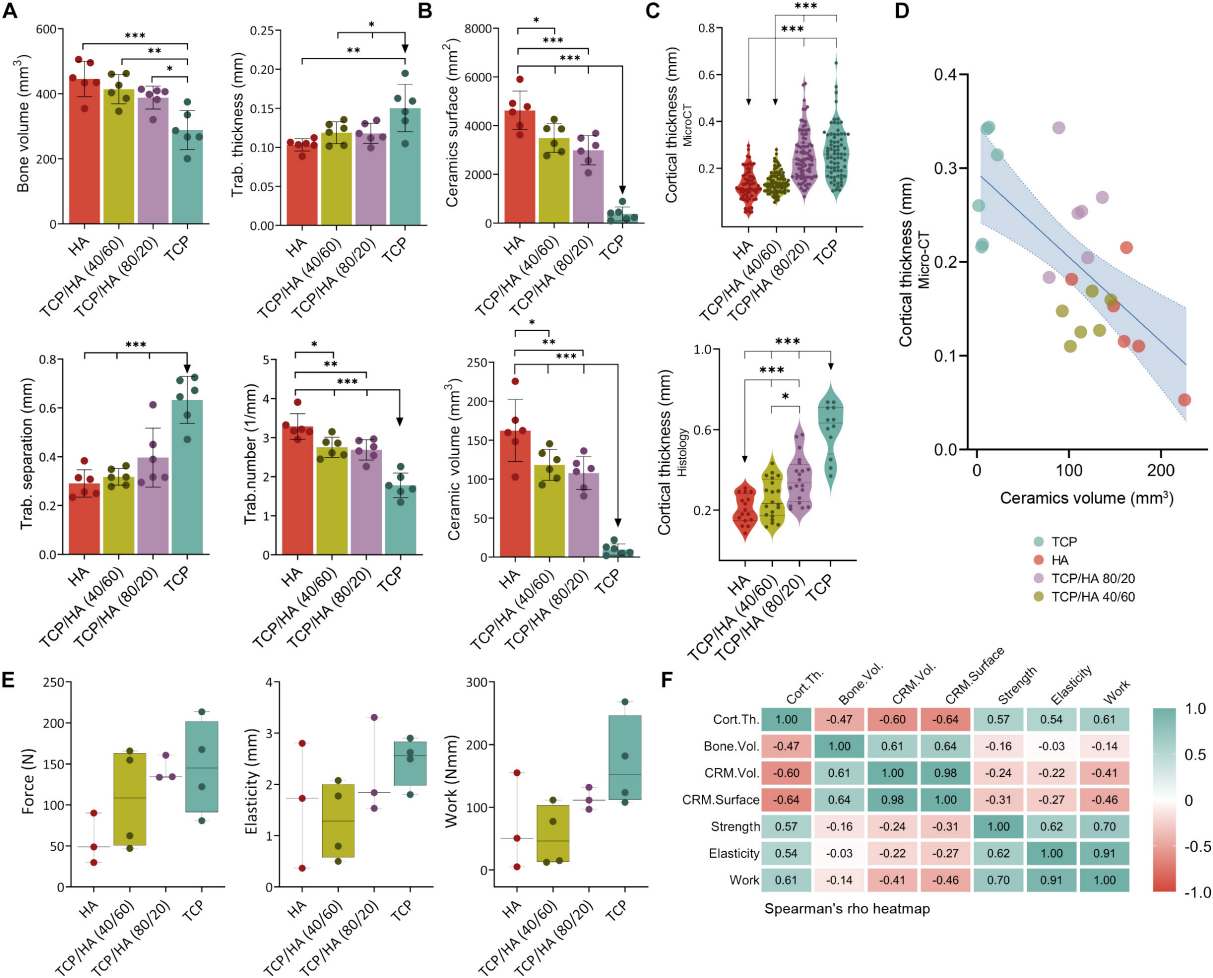
Micro-CT and biomechanical parameters of newly formed bone induced between 2 transverse processes in the rabbit PLF model by different Osteogrow-C formulations containing small ceramic granules of varying chemical composition. (A) Micro-CT quantification of bone volume (mm^3^), and trabecular parameters including thickness (mm), separation (mm), and number (1/mm). (B) Micro-CT quantification of volume (mm^3^) and surface area (mm^2^) of residual ceramic granules. (C) Cortical thickness (mm) was measured using both micro-CT imaging and histological sections. The number of specimens analyzed per group was 6. (D) Spearman’s rank pairwise correlation of micro-CT evaluated cortical thickness (mm) and CRM volume (mm^3^). (E) Biomechanical properties [force (N), elasticity (mm), and work (Nmm)] evaluated by the 3-point bending test (the number of analyzed specimens was 3 or 4 per group). (F) Spearman’s rank correlation heatmap displaying the relationships among micro-CT parameters and biomechanical properties. Strong positive (green) and negative (red) correlations are highlighted with color intensity. All statistically significant differences are annotated (*P ≤ 0.05, **P ≤ 0.01, and ***P ≤ 0.001).

Osteogrow-C implants in all experimental groups induced long-lasting spinal fusion, as observed through micro-CT, histological sections, and biomechanical evaluations conducted 365 d post-surgery (Figs. 6 and 7). The evaluation of micro-CT and histological sections revealed that the bone induced by Osteogrow-C implants was fully integrated with the native bone of the transverse processes, resulting in a solid fusion (Fig. 6A).

The primary difference among experimental groups was the amount of residual ceramics (Figs. 6 and 7B). Micro-CT analysis showed that TCP granules were almost completely resorbed, BCP granules were partially resorbed, while HA granules remained nearly intact (Fig. 7B). Histological analysis indicated that differences in ceramic resorption impacted the microarchitecture of the bone, fusing the adjacent transverse processes (Fig. 6B). In samples where fusion was induced using Osteogrow-C with ceramic granules composed of HA (Fig. 6B, first row) and BCP with a high proportion of HA (TCP/HA 40/60) (Fig. 6B, second row), minimally resorbed ceramic granules were observed. The newly formed bone in these samples consisted of discrete cortical bone and a central region where nonresorbed granules were connected by newly formed bone, with significantly less bone marrow compared to samples containing granules made of TCP and BCP with a high proportion of TCP. The bone architecture induced by Osteogrow-C with BCP containing a high proportion of TCP (TCP/HA 80/20) (Fig. 6B, third row) was similar to that observed with TCP ceramics (Fig. 6B, 4th row).

The main difference was the presence of some nonresorbed granules in specific parts of the implant. In samples where spinal fusion was achieved using Osteogrow-C with TCP ceramics, ceramic granules were almost entirely resorbed, and the newly formed bone consisted of well-defined cortical bone at the periphery and trabecular bone in the implant’s central regions. The trabecular bone was composed of trabeculae surrounded by bone marrow containing both hematopoietic cells and adipocytes. Micro-CT analysis showed that bone volume in the fusion mass was significantly greater in animals treated with Osteogrow-C implants containing HA and BCP ceramics than in those treated with pure TCP ceramics (Fig. 7A).

Additionally, trabecular number correlated with bone volume, with the lowest values observed in the TCP group (Fig. 7A). In contrast, trabecular thickness and separation were significantly higher in animals treated with Osteogrow-C containing TCP compared to those treated with BCP or pure HA ceramics (Fig. 7A). Residual ceramic volume and surface area are highest in HA and lowest in TCP, with biphasic ceramics showing intermediate properties (Fig. 7B). Cortical thickness, evaluated histologically and by micro-CT, showed that Osteogrow-C, containing TCP and TCP/HA 80/20, induced a bone fusion mass with thicker cortical bone, in comparison to HA and TCP/HA 40/60 (Fig. 7C). Furthermore, there is a negative correlation between ceramic volume and cortical thickness, suggesting that lower residual ceramics are associated with improved bone outcomes (Fig. 7D).

### Bone with a lower amount of residual ceramics has better biomechanical properties than bone with a higher amount

The biomechanical properties of the fusion mass were assessed using a 3-point bending test. Although the difference was not statistically significant, Osteogrow-C with TCP or BCP, containing a high proportion of TCP (TCP/HA 80/20), showed a trend toward superiority over Osteogrow-C containing HA or BCP with a high proportion of HA (TCP/HA 40/60) (Fig. 7E). Specifically, maximum force, elasticity, and work-to-break were higher in specimens containing TCP and BCP with an 80/20 TCP/HA ratio than in those containing HA and BCP with a 40/60 TCP/HA ratio. It is important to note that the biomechanical properties of samples containing granules composed of TCP or BCP with a high TCP content correlated with increased cortical thickness in those samples (Fig. 7F). Despite these differences, all specimens were biomechanically competent, regardless of ceramic composition.

## Discussion

To develop an optimal treatment for bone regeneration, we combined the osteoinductive rhBMP6, delivered in an ABC, with synthetic ceramics made of CaP, resulting in the development of the Osteogrow-C device. CaP ceramics as a component of Osteogrow-C implant not only play a crucial role as a CRM but also contribute with their excellent osteoconductive properties due to specific topography, resorption rate, and consequently ion release. Namely, the surface topography of CaP granules includes both the microscale and nanoscale features such as roughness, porosity, and geometric patterns that can influence cellular responses [35]. The distinct surface morphologies of CaP granules can arise from differences in crystal growth dynamics and sintering behavior [36]. These topographical characteristics can lead to possible osteoinductive properties [37,38] due to enhanced protein adsorption, facilitating osteoblast adhesion and subsequent differentiation [35,39]. Also, an interconnected porous network promotes cell infiltration and neovascularization, critical for nutrient transport and integration of new bone tissue [40]. CaP resorption rates, adjustable through composition variations, enable gradual scaffold degradation synchronized with new bone formation [3]. All these features contribute to the effectiveness of Osteogrow-C, enabling precise tailoring of the novel osteoinductive device.

In previous studies, it has been demonstrated that, following subcutaneous implantation in rats, Osteogrow-C induces the formation of vascularized ectopic composite ossicles composed of newly formed bone and residual ceramic granules [22,32]. Moreover, it has been shown that Osteogrow-C is both efficacious and safe in promoting PLF in rabbits [25,26] and sheep [24], as well as in healing large segmental defects in rabbits [23] and dogs [33]. However, the optimal chemical composition and size of ceramic granules to be used as a CRM in the Osteogrow-C device remained unclear. Unlike prior studies, which were largely short-term, this work pairs a 1-year follow-up with 2 complementary animal models to provide an integrated evaluation of long-term ceramic resorption and induced bone microarchitecture at both ectopic and functionally active sites. Specifically, we compared for the first time how granule chemical composition and size affect ceramic resorption and the structure of induced bone after 1 year at an ectopic site in rats and at a functionally active site employing the rabbit PLF model. Although alternative models for studying musculoskeletal diseases [41,42] have emerged in recent years, animal models remain essential for investigating surgical and regenerative interventions.

The first important finding of this study is that the ceramic granule size determines the bone volume and structural properties of the ectopic ossicles at the subcutaneous ectopic site, as evaluated 1 year after implantation. It has been previously found that small granules were superior to large granules at an earlier time point (day 21) in rats [22]. In this study, the differences were even more pronounced, with significantly higher bone formation in ectopic ossicles containing small granules (74 to 420 μm) in comparison to ectopic ossicles containing medium (500 to 1,700 μm) or large (2,360 to 4,000 μm) ceramic granules, regardless of chemical composition. Previous studies examining the influence of the chemical composition of CaP granules on BMP-induced bone volume have reported inconsistent findings [15–18]. In contrast, our study demonstrated that, in the subcutaneous assay, the effect of chemical composition is minimal compared to the pronounced impact of granule size. Furthermore, this study revealed that the bone structural patterns, determined by granule size and established during the first month following Osteogrow-C implantation [22], persisted over time. Thus, ectopic composite ossicles with small granules contained areas of compact-like bone and a dense trabecular network between the granules, whereas ossicles with medium and large granules showed few trabeculae and abundant bone marrow between adjacent ceramic granules. Despite their clear biological advantage in terms of bone formation, the practical application of small ceramic granules in Osteogrow-C implants presents formulation challenges. When used in limited amounts, small granules tend to cluster locally within the implant matrix, leading to regions depleted of ceramic content and uneven distribution. This issue may be alleviated by increasing the amount of small granules or by optimizing mixing and processing procedures to achieve more homogeneous dispersion. In contrast, larger granules tend to have more uniform distribution within the implant, which likely contributes to the superior biomechanical properties observed in Osteogrow-C implants containing granules larger than 500 μm. Consequently, although implants with small granules achieved the highest bone volume, larger granules may be more suitable as CRM for indications requiring larger implant volumes, such as large segmental bone defects [23,33].

A second key finding is that the chemical composition of ceramic granules dictates their resorbability, which varies considerably between the subcutaneous assay in rats and the functional site of the PLF model in rabbits. In our previous research involving a rat subcutaneous assay, due to the short end-point (21 d), there were no significant differences in the resorption of ceramics made from HA, β-TCP, and BCP (TCP/HA 80/20) [22]. The longer observation period in this study allowed us to demonstrate that ceramic granules composed of HA had a significantly lower resorption rate compared to TCP granules, while CRM resorption was less influenced by granule size. Nevertheless, a significant amount of ceramic material remained unresorbed in the ectopic composite ossicles at the subcutaneous site in rats. Conversely, in the rabbit PLF experiment, implants with different chemical compositions resulted in striking differences in the amount of residual ceramics. After 1 year, TCP granules were completely resorbed, suggesting a significantly higher CRM resorption rate at functionally active sites, such as spinal fusions, compared to the ectopic site in rats. On the other hand, HA granules remained relatively intact 1 year after implantation. This difference has direct structural consequences: Complete resorption of TCP granules allowed the newly formed bone to remodel into thicker cortices, while persistent HA particles constrained remodeling, maintaining a dense trabecular network tightly enveloping the nonresorbed ceramics. Consequently, Osteogrow-C implants containing HA produced a dense bone network surrounding the ceramic granules, while implants with TCP showed only trabeculae surrounded by abundant bone marrow. Finally, micro-CT analyses revealed that bone volume was significantly higher in implants containing HA granules compared to those with TCP. Thus, we suggest that at functionally active sites, where significant resorption of ceramic granules takes place, the chemical composition of these granules plays a crucial role in determining both the quantity and structural characteristics of the bone. We hypothesize that the observed differences arise because the rabbit PLF site is a surgically decorticated, well-vascularized bone bed enriched with progenitor cells and cytokine gradients that drive remodeling [43], whereas rat subcutaneous pockets are ectopic, less vascularized, and dominated by a foreign body response [44]. Namely, PLF site biology possibly yields higher osteoclast-mediated resorption and osteoclast/foreign body giant cell (FBGC) activity in comparison to the subcutaneous site. Furthermore, the resorption of ceramic particles results in the substantial release of calcium (Ca^2+^) and phosphate (PO₄^3−^) ions, which may play an important role in both osteogenesis and the immune response. Specifically, calcium ions can promote the osteogenic differentiation of mesenchymal stem cells, whereas phosphate ions enhance osteoblast mineralization and alkaline phosphatase (ALP) activity. However, phosphate ions may also induce pro-inflammatory M1 macrophage polarization, leading to foreign body reactions [45]. It is also worth noting that particle size and distribution can influence the release profiles of calcium and phosphate ions. In particular, the higher surface area-to-volume ratio of smaller particles enhances calcium ion release, promoting the fusion of adherent macrophages [46]. Another possible contributing factor is the presence of muscle-driven micromotion and interstitial fluid flow, which can enhance cell recruitment and fluid shear-modulated remodeling [47], although these mechanical effects are likely secondary to the local biological milieu. It is worth noting that the rabbit PLF model is not a truly load-bearing site, in comparison to humans, where the mechanical load would be significantly higher. Lastly, it is essential to note that species differ in innate immune kinetics, macrophage polarization, FBGC formation, and bone turnover, which may also contribute to the variation in apparent degradation rates for the same material. Collectively, these findings establish an integrated mechanistic link between ceramic size and composition, their resorption behavior, and the ensuing bone architecture.

The third important finding of this study is that bone with complete (TCP group) or significant (TCP/HA 80/20 group) resorption of ceramic granules exhibits better biomechanical properties compared to bone containing a large amount of nonresorbed granules (HA and TCP/HA 40/60 groups). These superior biomechanical properties can be attributed to the significantly thicker and more pronounced cortical bone in the groups where ceramic resorption occurred, as opposed to those containing nonresorbed granules. This outcome highlights the distinction between bone quantity and bone quality: Although the HA group had the highest bone volume due to the dense trabecular network around persistent granules, its limited remodeling capacity led to thin cortices and lower mechanical strength. In contrast, faster-resorbing TCP granules led to lower total bone volume but enabled the formation of thicker cortices. The results of this study suggest that cortical bone thickness is the primary determinant of the biomechanical properties of newly formed bone, given that the total bone volume and number of trabeculae are greater in groups containing nonresorbed ceramic granules (HA and TCP/HA 40/60 group). These findings are consistent with our previous studies on the PLF model in rabbits [25] and sheep [24], where it has been shown that an increase in cortical bone thickness over time correlates with improved biomechanical properties.

Carriers that are resorbed quickly after implantation are usually compressible and have unsatisfactory biomechanical properties for use in applications where compressive forces are present [48,49]. In previous studies, it has been demonstrated that the addition of ceramic granules to ABC as a carrier for BMPs significantly improves spinal fusion outcomes [24–26]. However, previous research left an important question unanswered—defining what the optimal chemical composition of ceramic granules is for long-term outcomes on functionally active sites, and how the resorption rate impacts the biomechanical properties of the newly formed bone [24–26]. In this study, we have demonstrated that ceramic resorption is strongly enhanced on a functionally active site. We speculate that this may be due to the presence of osteoclast precursors and cytokine gradients, or through mechanotransduction pathways, which could promote remodeling and replacement with newly formed bone. In ectopic environments, such events are likely diminished or absent, resulting in limited or inefficient remodeling. Consequently, the persistence of ceramic material in these sites may be primarily attributed to its slow passive dissolution rather than active, cell-mediated resorption.

In conclusion, this study provides the first comprehensive evaluation of how ceramic granule size and chemical composition in different Osteogrow-C formulations govern long-term bone microarchitecture and ceramic resorption in 2 distinct animal models, with evaluation 1 year after implantation. Although the ceramics were tested within Osteogrow-C implants, the principles identified are likely applicable to other ceramic-based carriers used in bone regeneration. In the rat subcutaneous model, ceramic composition played only a minor role, as this low-load, low-remodeling environment favors rhBMP6-driven osteoinduction governed primarily by implant microstructure—particularly granule size (surface area), packing density, and pore architecture—over composition-dependent resorption or ion release. In contrast, in the rabbit PLF model—where sustained loading and remodeling are critical—chemical composition strongly dictated resorption kinetics and the resulting bone architecture. Namely, TCP-based Osteogrow-C consistently provided the most favorable functional outcome: complete (or near-complete) granule resorption, which enabled robust remodeling and thicker cortical bone, translating into superior biomechanical performance despite lower bone volume than HA-based Osteogrow-C formulations, which tended to retain granules and yield higher bone volume but mechanically inferior, less remodeled bone. Collectively, these findings support TCP, or biphasic ceramics with a high TCP fraction, as the preferred ceramic strategy when the goal is mechanically competent, well-remodeled, BMP-induced bone at clinically relevant fusion sites.

## Data Availability

Raw data were generated at the Laboratory for Mineralized Tissues. Derived data supporting the findings of this study are available from the corresponding author, S.V., upon request.
