# Supplementary material for: Bone Induction as a Function of Size and Chemical Composition of Calcium Phosphate Granules in Osteogrow-C Evaluated in Animal Models: A 1-Year Follow-Up
Source: Biomater Res. 2026 Feb 25;30:0337. doi: 10.34133/bmr.0337 (PMC12932868; doi:10.34133/bmr.0337)
Supplement: Supplementary 1 — Fig. S1 [file bmr.0337.f1.docx]

***
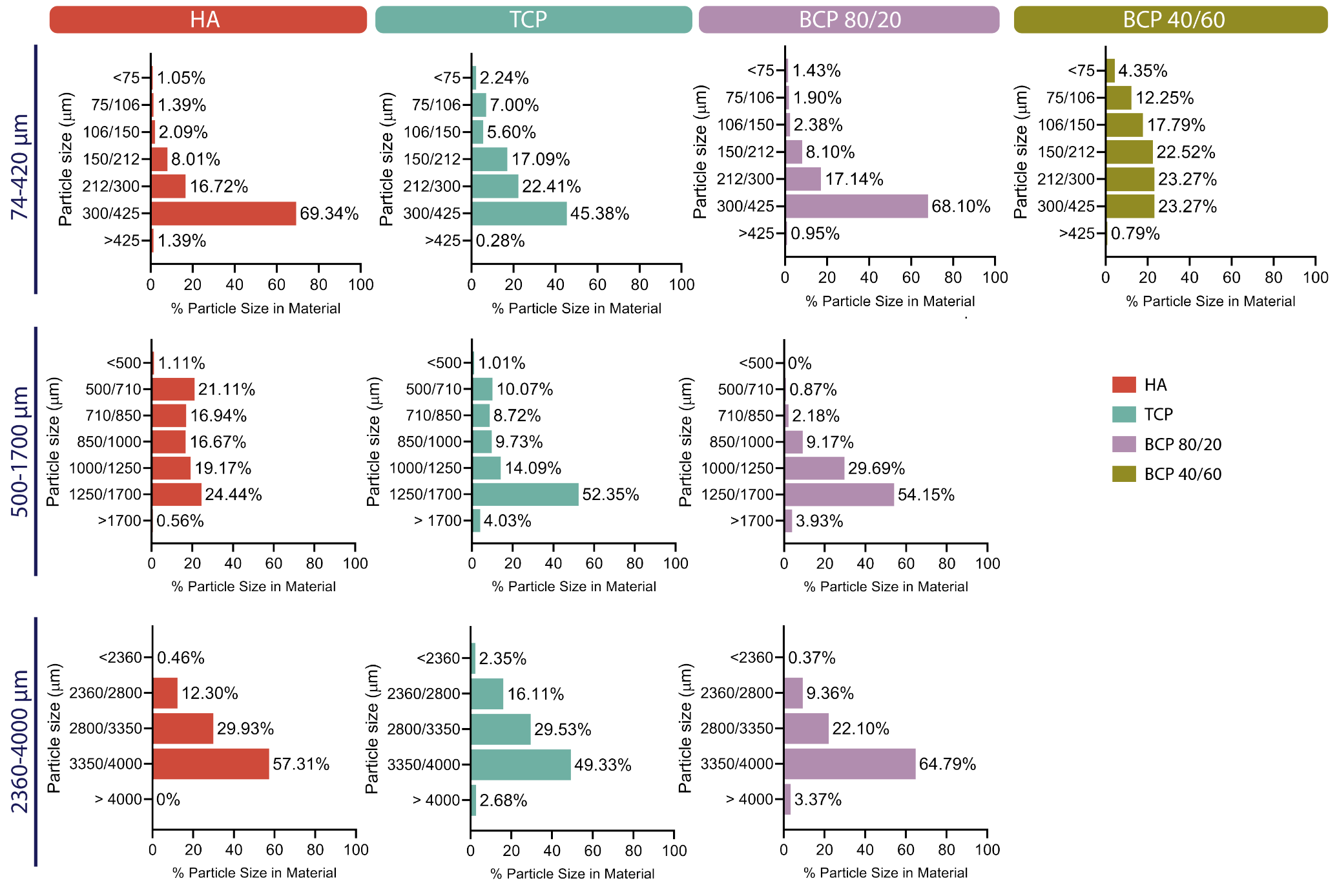
Supplement 1****. Sieve-based particle-size distribution for HA, β-TCP, BCP 80/20, and BCP 40/60 granulates, presented as % of total mass per sieve interval.*
